# A Method for Generation of Bone Marrow-Derived Macrophages from Cryopreserved Mouse Bone Marrow Cells

**DOI:** 10.1371/journal.pone.0015263

**Published:** 2010-12-17

**Authors:** Fernanda M. Marim, Tatiana N. Silveira, Djalma S. Lima, Dario S. Zamboni

**Affiliations:** Department of Cell Biology, School of Medicine of Ribeirão Preto, University of São Paulo (FMRP/USP), Ribeirão Preto, Brazil; Fundação Oswaldo Cruz, Brazil

## Abstract

The broad use of transgenic and gene-targeted mice has established bone marrow-derived macrophages (BMDM) as important mammalian host cells for investigation of the macrophages biology. Over the last decade, extensive research has been done to determine how to freeze and store viable hematopoietic human cells; however, there is no information regarding generation of BMDM from frozen murine bone marrow (BM) cells. Here, we establish a highly efficient protocol to freeze murine BM cells and further generate BMDM. Cryopreserved murine BM cells maintain their potential for BMDM differentiation for more than 6 years. We compared BMDM obtained from fresh and frozen BM cells and found that both are similarly able to trigger the expression of CD80 and CD86 in response to LPS or infection with the intracellular bacteria *Legionella pneumophila*. Additionally, BMDM obtained from fresh or frozen BM cells equally restrict or support the intracellular multiplication of pathogens such as *L. pneumophila* and the protozoan parasite *Leishmania (L.) amazonensis*. Although further investigation are required to support the use of the method for generation of dendritic cells, preliminary experiments indicate that bone marrow-derived dendritic cells can also be generated from cryopreserved BM cells. Overall, the method described and validated herein represents a technical advance as it allows ready and easy generation of BMDM from a stock of frozen BM cells.

## Introduction

Macrophages are essential for both the innate and adaptive immune system, as they play key roles in different biological processes, such as antigen presentation and processing, microbial killing, cytokine production, and clearance of apoptotic cells, among others [1], [2], [3]. Consequently, murine macrophages have become an important host cell model for investigation of mammalian macrophage functions. Therefore, there is great demand for a homogeneous macrophage population on which to conduct such immunological investigations. Because differentiated primary macrophages display limited multiplication, several investigators have used immortalized macrophage-like myeloid cell lines, such as J774A.1, RAW264.7 and P388D1. However, these cells usually differ from primary macrophages, as the selective pressure imposed by continual subculture usually results in the loss of genes that are not important for multiplication yet are key for macrophage immune functions.

Regardless of the deficiencies of macrophage-like cells, the broad use of transgenic and gene-disrupted mice has emphasized the need for cultures of primary cells explanted from these animals. Currently, the three major choices for primary mouse macrophages are peritoneal macrophages (PM), alveolar macrophages (AM) and bone marrow-derived macrophages (BMDM). Although resident PM and AM can be readily harvested from the mouse, the yield is low and the macrophages' biology is severely affected by the sanitary conditions of the animal facility. In contrast, BMDM are fully differentiated *in vitro* from bone marrow (BM) stem cells and therefore are naïve regardless of the health conditions of the donor mice. In addition, high numbers of BMDM can be obtained from a single mouse. These features have favored the choice of BMDM as a macrophage model in most immunological studies.

Over the last decade, methods to freeze and store viable hematopoietic human cells have been extensively investigated [4], [5], [6], [7]. However, to the best of our knowledge, there is no information related to how to prepare murine BMDM from frozen BM cells. Here, we establish a highly efficient protocol for freezing murine BM cells and further generating BMDM. We further compared BMDM obtained from fresh or previously frozen BM cells and found that both BMDM are similarly able to express stimulatory and co-stimulatory molecules in response to inflammatory stimuli and to respond to infection by the intracellular bacterial pathogen *Legionella pneumophila*.

## Materials and Methods

### Preparation of murine bone marrow cells

The protocols for animal handling were previously approved by our institutional Animal Ethics Committee (CETEA/FMRP, protocol number 006/2008). Femurs were obtained from 6–12 week old C57BL/6 mice. After euthanasia, the mice were sprayed with 70% ethanol and the femurs were dissected using scissors, cutting through the tibia below the knee joints as well as through the pelvic bone close to the hip joint. Muscles connected to the bone were removed using clean gauze, and the femurs were placed into a polypropylene tube containing sterile PBS on ice. In a tissue culture hood, the bones were placed in 70% ethanol for 1 minute, washed in sterile RPMI 1640 and then both epiphyses were removed using sterile scissors and forceps. The bones were flushed with a syringe filled with RPMI 1640 to extrude bone marrow into a 15 mL sterile polypropylene tube. A 5 ml plastic pipette was used to gently homogenize the bone marrow. The cell suspension generated thereafter is called fresh bone marrow cells.

### Freezing and thawing bone marrow cells

Fresh bone marrow cells were counted using a hemocytometer, centrifuged for 5 minutes at 200*× g* at 4°C and gently resuspended to obtain a solution containing from 4 to 6×10^6^ cells/ml (unless otherwise mentioned) in freezing media containing 90% fetal bovine serum and 10% DMSO (unless otherwise mentioned). Each milliliter of the solution was added to an individual cryovial, maintained in a −80°C freezer for 24 hours and then transferred to a liquid nitrogen tank. To thaw the cells, a cryovial was quickly transferred to a 37°C water bath until the suspension was completely thawed. The contents were then transferred to plastic tubes containing 10 ml of 37°C PBS. The cells were centrifuged at 200*× g* for 5 minutes and resuspended in bone marrow differentiation media as described below.

### Differentiation of bone marrow-derived macrophages

Fresh or frozen bone marrow cells were used to generate BMDM as previously described [8], using L929-cell conditioned medium (LCCM) as a source of granulocyte/macrophage colony stimulating factor [9]. The cells were resuspended in 10 ml bone marrow differentiation media (R20/30), which is RPMI1640 supplemented with 20% fetal bovine serum (Gibco, cat. 12657-029), 30% LCCM, 100 U/ml penicillin, 100 µg/ml streptomycin, and 2 mM L-glutamine. Cells were seeded in non-tissue culture treated Optilux Petri dishes (BD Biosciences) and incubated at 37°C in a 5% CO_2_ atmosphere. Four days after seeding the cells, an extra 10 ml of fresh R20/30 were added per plate and incubated for an additional 3 days. To obtain the BMDM, the supernatants were discarded and the attached cells were washed with 10 ml of sterile PBS. Ten ml of ice-cold PBS were added to each plate and incubated at 4°C for 10 minutes. The macrophages were detached by gently pipetting the PBS across the dish. The cells were centrifuged at 200*× g* for 5 minutes and resuspended in 10 ml of BMDM cultivation media (R10/5), which is composed of RPMI 1640, 10% fetal bovine serum, 5% LCCM and 2 mM L-glutamine. The cells were counted, seeded and cultivated in tissue culture plates 12 hours before any further experimental procedure.

### Bacterial infection

*Legionella pneumophila* Lp01 [10] and isogenic mutants deficient for flagellin (*flaA*) [11] were used in this study. Bacteria were grown on charcoal yeast extract agar (BCYET: 1% yeast extract, 1% N-(2 acetoamido)-2-aminoethaneosulfonic (ACES) pH 6.9, 3.3 mM L-cysteine, 0.33 mM Fe(NO)_3_, 1.5% bacto agar and 0.2% activated charcoal, supplemented with 100 mg/ml thymidine) at 37°C for 48 hours as previously described [12]. For infections, bacteria were resuspended in sterile water and diluted to an appropriate multiplicity of infection (MOI) based on optical density (OD) at 600 nm. *L. pneumophila* growth curves in BMDM was assessed as previously described [13]. Briefly, BMDM were added to 24-well plates at a density of 2×10^5^ cells per well and wild type *L. pneumophila* or *flaA* were added to each well at an MOI of 0.015. Macrophages were lysed in sterile water after 48 hours of infection and the cell lysates were combined with the tissue culture supernatant for each respective well, ensuring that all bacteria in the well would be counted. Serial dilutions from each well were plated on BCYET plates. Colony forming units were assessed by counting the bacterial colonies present after 96 hours of incubation at 37°C. For determination of the frequency of bacteria per LCV, BMDMs were seeded at 2×10^5^ cells/well on 13-mm glass coverslips and cultivated in 24-well tissue culture dishes. The cells were infected with *L. pneumophila* with an MOI of 10 followed by centrifugation at 200× g for 5 minutes and incubation for 1 hour to allow phagocytosis. The cells were further washed 3 times with PBS and incubated for an additional of 4 or 6 hours. The cultures were fixed and permeabilized for 10 minutes in ice-cold methanol. Coverslips were washed with PBS and blocked in PBS containing 5% BSA and 2% goat serum for 1 hour. Bacteria were stained with a rabbit anti-*L. pneumophila* polyclonal antibody (1∶2000), followed by Alexa-488-conjugated goat anti-rabbit secondary antibody (1∶300 dilution, Invitrogen) and analyzed in a Leica TCS SP2 SE inverted microscope using a 40x/1.25 oil objective (Leica HCX PL APO).

### Flow cytometry

For flow cytometry experiments, BMDM and BMDCs were seeded and cultivated in non-tissue culture-treated 6-well plates at a density of 1×10^6^ cells per well. Cultures were treated with 1 µg/ml LPS (Escherichia coli 055:B5 LPS, Sigma) or infected with wild type *L. pneumophila* at an indicated MOI. After 18 hours incubation, macrophages and dendritic cells were removed from the plates with ice-cold PBS and stained with the following antibodies: PE-Cy7 anti-mouse CD11c (BD Pharmingen, clone HL3), APC anti-mouse MHC-II (eBioscience, clone M5/114.15.2), FITC anti-mouse CD86 (BD Pharmingen, clone GL-1), PE anti-mouse CD40 (eBioscience, clone 1C10), APC-Cy7 anti-mouse CD11b (BD Pharmingen, clone M1/70) and PercP anti-mouse F4/80 (eBioscience, clone BM8). The isotype controls were rat IgG2a (R35-95), rat IgG2b (A95-1), and hamster IgG2 (Ha4/8), all from BD Pharmingen. For stem cells staining, fresh and cryopreserved bone marrow cells were added to FASC tube at a density of 1×10^6^ cells per tube. Cells were staining with phycoerythrin (PE)-conjugated antibodies against: CD29 (eBioHMb1.1); CD34 (RAM34); CD44 (IM7); CD45 (30-F11); CD90 (53-2.1); CD105 (MJ7/18); Sca-1 (D7); PDGF (APB5); CD31 (BD cat. 553373); CD73 (BD cat. 550741) and isotype control (eB149/10H5) from BD Pharmingen. Briefly, cells were washed, incubated with an anti-FcRIII/II mAb (2-4G.1) in PBS containing 0,1% BSA and 0,01% NaN_3_ and stained for 30 minutes at 4°C. Data were acquired with a FACSCantoII (Benton Dickinson) and analyzed with FlowJo Software (Tree Star).

### *Leishmania amazonensis* infection

Promastigotes of the *L. amazonensis* constitutively expressing GFP (La-GFP; MHOM/BR/73M2269) were kindly provided by Dr. Silvia R. Uliana (ICB/USP). Parasites were cultured in Schneider's Drosophila medium (Invitrogen, Carlsbad, CA), pH 7.0, supplemented with 20% heat-inactivated fetal calf serum (GIBCO BRL), 100 U/ml penicillin G potassium (USB Corporation, Cleveland, OH, USA), 2 mM L-glutamine and 2% human urine, pH 6.5, at 26°C. BMDM were harvested and infected with La-GFP at 10∶1 parasite-to-cell ratio. After 6 h of infection, extracellular parasites were washed and fresh media were added. The percentage of GFP^+^ BMDM and the GFP MIF was determined after 48 h of infection by flow cytometry [14]. Damaged cells were revealed by staining cells with 10 µg/ml solution of propidium iodide (PI; Sigma) added 5 minutes before the FACS measurements.

### Generation of Dendritic cells

Dendritic cells (DC) were generated in vitro from bone marrow cells from 6- to 8-wk-old wild type C57BL/6 as previously described [15]. Briefly, femurs and tibias were flushed with RPMI-1640 (Gibco-BRL Life Technologies, Grand Island, NY) to release the BM cells that were cultured in 24-well-culture plates in RPMI-1640 (Gibco) supplemented with 10% heat-inactivated FCS, 100 µg/ml of penicillin, 100 µg/ml of streptomycin, 5×10^−5^ M 2-mercaptoethanol (all from Sigma), 20 ng/ml murine granulocyte-macrophage colony-stimulating factor (GM-CSF, Invitrogen). On day 3, the supernatants were gently removed and replaced with the same volume of media. On day 6, the non-adherent cells were collected and submitted to a positive selection using anti-CD11c magnetic beads, according to the manufacturer's instructions (Miltenyi Biotec). Flow cytometry evaluation of purified DC shows that more than 91% of cells were CD11c positive.

## Results

### Determination of the optimal DMSO concentration and numbers of BM cells

**Figure 1:**
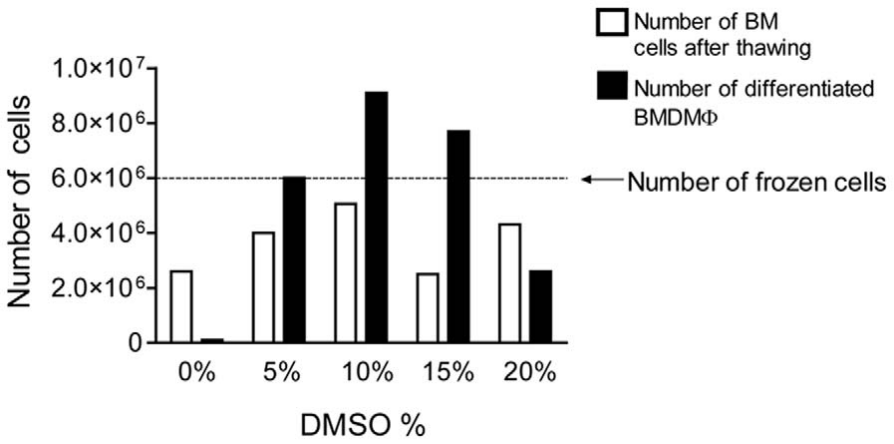
Determination of the optimal DMSO concentration for cryopreservation of bone marrow cells. Bone marrow (BM) cells were resuspended in heat-inactivated fetal bovine serum containing 0, 5, 10, 15 or 20% DMSO and then stored in liquid nitrogen for one week. Cells were thawed and counted to estimate the number of BM cells after thawing (white bars) and were then cultivated in bone marrow differentiation media. After 7 days of differentiation, the cultures were washed with ice cold PBS to remove undifferentiated cells and the bone marrow derived macrophages (BMDM) were detached from the plates and counted (black bars). The dotted line represents the initial number of frozen BM cells per aliquot.

**Figure 2:**
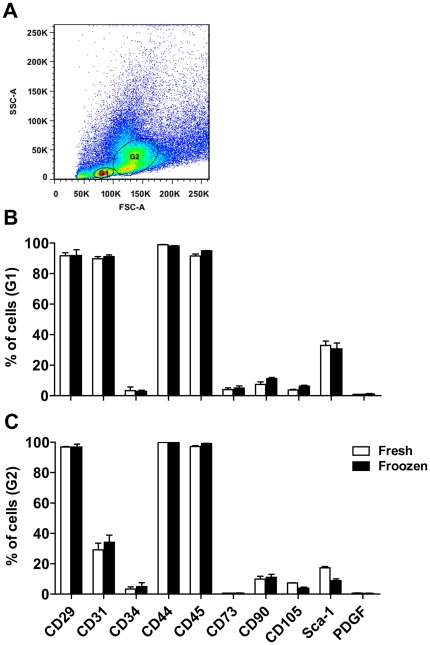
Fresh or cryopreserved bone marrow cells show similar expression of cell markers. Cells from fresh or cryopreserved (frozen) bone marrow were harvested and labeled with phycoerythrin (PE)-conjugated Abs against CD29, CD31, CD34, CD44, CD45, CD73, CD90, CD105, Sca-1 and PDGF or control IgGs and analyzed by FACS. (A) Dot plot showing the two gates analyzed (G1 and G2). (B and C) Averages and standard deviation of the triplicates show the percentages of the cells positive for each marker within the gated population: G1 (B) or G2 (C). Data are one representative experiment out of two independent experiments performed. No statistically significant differences were detected between fresh or frozen BM cells (*P*>0.05).

Cryopreservation methods usually use dimethyl sulfoxide (DMSO) to prevent the formation of ice crystals that damage membranes [16]. Because the DMSO concentrations vary among the protocols for freezing immortalized cell lines, we first investigated the optimal DMSO concentration to be used in a protocol to freeze bone marrow cells, which should remain viable to allow further generation of BMDM. We initially used 3×10^7^ cells from a single mouse and divided them into 5 identical aliquots containing 6×10^6^ cells each. These aliquots were then resuspended in fetal bovine serum containing 0, 5, 10, 15 and 20% DMSO and were stored in liquid nitrogen for one week. After thawing, we estimated the numbers of viable bone marrow cells per frozen vial and found that cells that were frozen in 90% FBS +10% DMSO yielded the highest recovery of intact cells (Fig. 1, white bars). We further used these thawed cells to generate bone marrow derived macrophages and found that the highest recovery of differentiated BMDM was obtained when 10% DMSO was used to freeze the bone marrow cells (Fig. 1, black bars). Collectively, these data indicate that viable BM cells can be maintained frozen, and once thawed, they can readily be used to generate BMDM. The solution containing 10% DMSO in 90% fetal bovine serum seems most appropriate for the cryopreservation (Fig. 1). To further investigate if the freezing process differentially affects the viability of BM cell populations, we used a several surface markers of BM cells to evaluate the percentages of specific cell populations before and after the freezing process. As shown in figure 2, the percentages of BM cells expressing CD29, CD31, CD34, CD44, CD45, CD73, CD90, CD105 and Sca-1 was not altered in fresh of cryopreserved BM cells, thus indicating that freezing protocol may not differentially affect specific BM populations.

**Figure 3:**
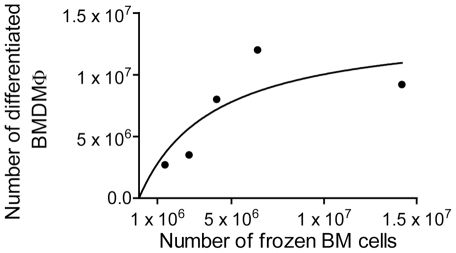
Numbers of frozen bone marrow cells per differentiated bone marrow-derived macrophages. Bone marrow cells (BM) were obtained from a single mouse and frozen at concentrations of 1.4×10^6^, 2.7×10^6^, 4.2×10^6^, 6.4×10^6^ or 1.4×10^7^ cells/vial. The cells were thawed and cultivated in bone marrow differentiation media for 7 days. The cultures were washed with ice cold PBS to remove undifferentiated cells and the bone marrow derived macrophages (BMDM) were detached from the plates and counted. The figure shows the non-linear correlation between the number of frozen BM cells (x-axis) and the number of differentiated BMDM (y-axis).

Next, we investigated the optimal number of bone marrow cells to be added to each cryovial in order to recover the maximum number of BMDM. Thus, we obtained cells from a single mouse and froze 1.4×10^6^, 2.7×10^6^, 4.2×10^6^, 6.4×10^6^ or 1.4×10^7^ BM cells per cryovial. The vials were further thawed and the cells were differentiated into BMDM. When we used low numbers of frozen BM cells per cryovial, we found a direct correlation between the number of frozen BM cells and the number of differentiated BMDM recovered (Fig. 3). However, when we added higher numbers of cells, i.e., 5×10^6^ BM cells, we obtained less BMDM than expected in a linear regression. It is possible that differentiation efficiency was decreased due to saturation of the nutrients in the media. Based on the data shown in Figure 3, we estimate that addition of 4 to 6×10^6^ BM cells/vial is appropriate for BMDM generation.

### Frozen BM cells maintain differentiation capacity for at least 6 years

**Figure 4:**
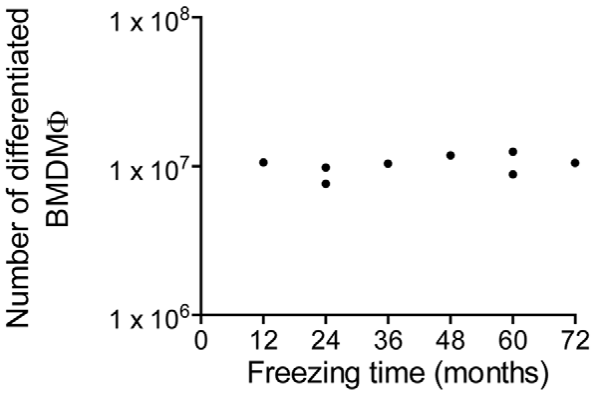
Frozen bone marrow cells maintain differentiation competence over time. Bone marrow cells were frozen at a concentration of 4×10^6^ cells per vial at different times and maintained in liquid nitrogen. Eight aliquots were simultaneously thawed and the cells were cultivated in bone marrow differentiation media. After 7 days of differentiation, the cultures were washed with ice cold PBS to remove undifferentiated cells and the bone marrow derived macrophages (BMDM) were detached from the plates and counted.

The freezing method described herein is very useful as it allows for the storage of viable BM cells that can be used for further generation of BMDM. Therefore, it is important to know how long the frozen BM cells can retain differentiation competence. Thus, over the past 6 years, we have frozen BM cells at a concentration of 4×10^6^ cells/vial in a 10% DMSO, 90% FBS solution and maintained them in liquid nitrogen. In a single experiment, we thawed 8 of these vials and evaluated competence for BMDM differentiation. Over 6 years of storage, we did not detect any loss in differentiation capacity (Fig. 4). Although we did not calculate the differentiation ability for BM cells frozen for over 6 years, we believe that these cells can remain competent for differentiation for even longer periods, as previously reported for cryopreserved human stem cells [17].

### BMDM obtained from fresh or frozen BM cells trigger equal expression of CD80 and CD86 in response to inflammatory stimulus

**Figure 5:**
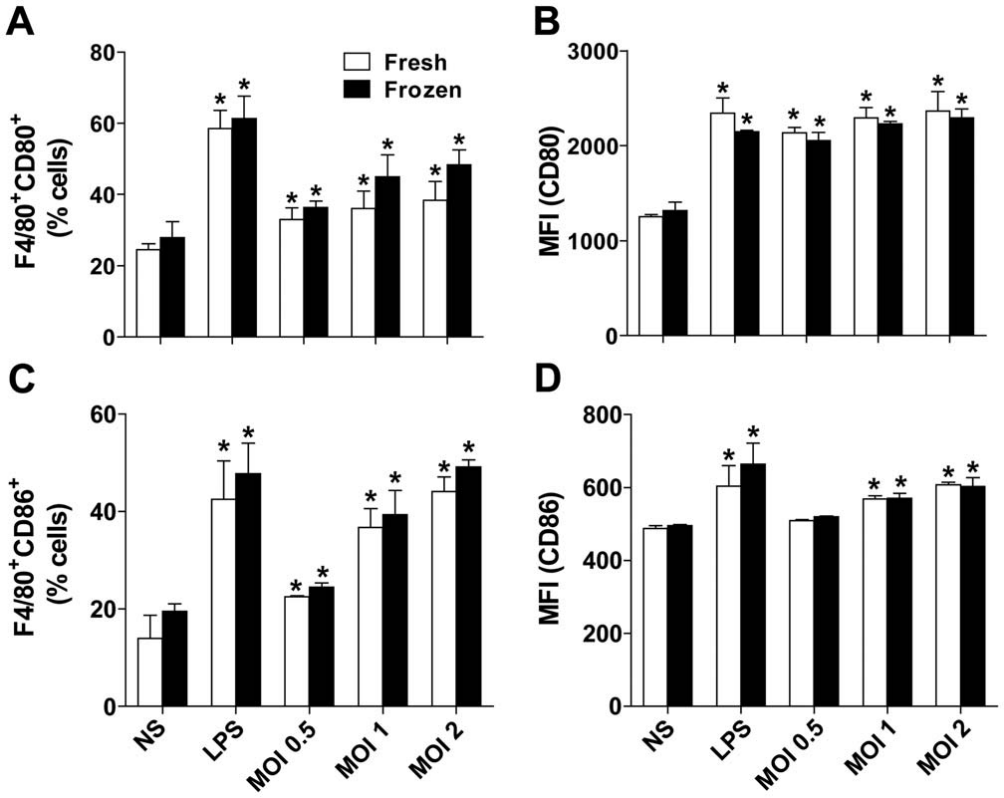
Bone marrow-derived macrophage obtained from fresh or cryopreserved bone marrow cells similarly express CD80 and CD86 in response to LPS and *Legionella pneumophila*. BMDMs obtained from fresh or cryopreserved (frozen) bone marrow cells were left untreated (NS), stimulated with 1 µg/ml LPS or infected with *L. pneumophila* at an MOI of 0.5, 1 and 2. Cultures were cultivated in non-tissue culture-treated 6 well-plates at a density of 1×10^6^ BMDM per well and stimulated/infected for 18 hours. Cells were stained for CD11b, F4/80, CD80 and CD86 and evaluated by flow cytometry. Plots were gated on CD11b^+^ cells. Shown are the percentage of cells F4/80^+^CD80^+^ (A) and F4/80^+^CD86^+^ (C). The mean fluorescence intensities are shown for CD80 (B) and CD86 (D). Data are representative of those found in three independent experiments. Asterisks indicate statistically significant differences in relation to NS (*P*<0.05). No statistically significant differences were detected between BMDM obtained from fresh or frozen BM cells (*P*>0.05).

Further investigation of the biological functions of BMDM obtained from frozen BM cells is required for routine use of this protocol. Therefore, we investigated important macrophage functions, such as expression of stimulatory molecules and the ability to restrict or support the multiplication of pathogenic microbes [3]. Thus, we aimed to compare the BMDM obtained from fresh or frozen BM cells with respect to expression of surface molecules, such as CD80 and CD86. For this comparison, BMDM were either stimulated with *E. coli* LPS or with the intracellular bacteria *Legionella pneumophila* and expression of CD80 and CD86 was assessed by FACS. As shown in Figure 5, BMDM readily activate expression of CD80 and CD86 in response to LPS or *L. pneumophila* infection. Importantly, BMDM obtained from fresh or frozen BM cells did not differ in expression of these molecules either in response to LPS or live *L. pneumophila* (Fig. 5).

### Dendritic cells can be generated from cryopreserved BM cells and equally trigger expression of CD40, CD86 and MHC-II in response to LPS

**Figure 6:**
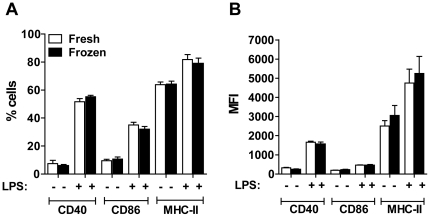
Bone marrow-derived dendritic cells obtained from fresh or cryopreserved bone marrow cells showed similar expression of CD40, CD86 and MHC-II in response to LPS. Bone marrow-derived dendritic cells (BMDCs) obtained from fresh or cryopreserved (frozen) bone marrow cells were either left untreated or stimulated with 1 µg/ml LPS for 24 h. BMDCs were stained for CD11b, CD11c, CD40, CD86 and MHC-II and analyzed by flow cytometry. Plots were gated on CD11b^+^ cells. Shown are the percentages (A) and mean fluorescence intensities (B) of CD11c^+^ cells expressing CD40, CD86 or MHC-II in response to LPS treatment. Data are representative of those found in two independent experiments. No statistically significant differences were detected between BMDCs obtained from fresh or frozen BM cells (*P*>0.05).

Although the aim of our study was to evaluate generation and functions of BMDMs from cryopreserved BM cells, we also preformed preliminary studies to assess the application of the method for generation of bone marrow-derived dendritic cells (BMDCs). We found that cryopreserved BM can effectively be differentiated to DCs in presence of GM-CSF. Furthermore, we found that BMDCs obtained from fresh or frozen BM cells equally trigger the expression of molecules such as CD40, CD86 and MHC-II in response to LPS stimulation (Fig. 6).

### BMDM obtained from fresh or frozen BM cells equally restrict and support the multiplication bacterial pathogen *L. pneumophila* and the protozoan parasite *L. amazonensis*

**Figure 7:**
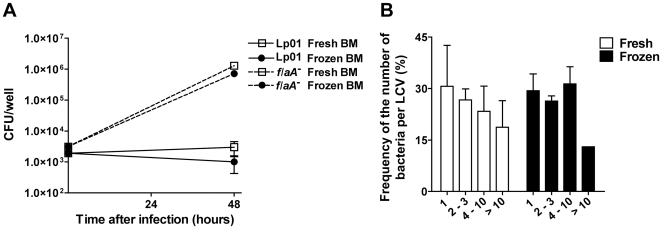
Bone marrow-derived macrophages obtained from fresh or frozen bone marrow cells respond similarly to infection with *Legionella pneumophila*. (A) Bone marrow-derived macrophages (BMDM) obtained from fresh (open squares) or cryopreserved (frozen; closed circles) bone marrow (BM) cells were infected with wild type *L. pneumophila* (Lp01, continuous lines) or with flagellin-deficient *L. pneumophila* (*flaA* ^-^, dashed lines). BMDM were cultivated and infected in 24-well plates with the indicated *Legionella* strain at an MOI of 0.015. After 48 hours post-infection, cells were lysed and the colony-forming units (CFU) were estimated for each of the triplicate wells. (B) Fresh (white bars) or frozen (black bars) BMDMs were infected with Lp01 at an MOI of 10 for 1 hour. Cultures were vigorously washed with PBS and further incubated for a total of 9 hours of infection. Bacteria were stained with a rabbit anti-*L. pneumophila* polyclonal antibody and the number of bacteria per individual LCV were enumerated using an epifluorescence microscope. Shown are the frequencies of LCV containing: 1; 2–3; 4–10; and more than 10 bacteria (>10). At least 100 LCV in independent cells were scored per each of the triplicate coverslips. Data are representative of those found in three (A) or two (B) independent experiments. No statistically significant differences were detected between BMDM obtained from fresh or frozen BM cells (*P*>0.05).

**Figure 8:**
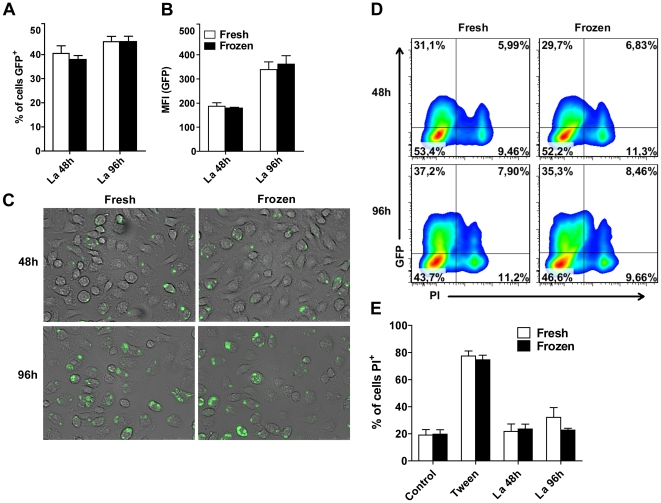
Bone marrow-derived macrophage obtained from fresh or cryopreserved bone marrow cells respond similarly to the infection with *Leishmania amazonensis*. Bone marrow-derived macrophages (BMDM) obtained from fresh or cryopreserved (frozen) bone marrow cells were infected with *L. amazonensis* promastigotes constitutively expressing the GFP at an MOI of 10 parasites per cell. After 48 h and 96 h of infection, the percentages of *L. amazonensis*-GFP^+^ and propidium iodide-positive cells were estimated by flow cytometry. (A) Percentage of GFP^+^ BMDM obtained from fresh (white bars) and frozen (black bars) BM cells. (B) Mean fluorescence intensities (MIF) for GFP signal. (C) Representative images of cultures infected for 48 h e 96 h with *L. amazonensis* GFP. (D) FACS profiles of BMDM obtained from fresh and frozen BM cells infected with *L. amazonensis* GFP. Shown are the percentages of cells with signal for GFP and PI. (E) Bar graphics indicating the percentage of propidium iodide-positive cells in uninfected cultures (control), cells treated with 0.05% Tween 20 (Tween) or after infection with *L. amazonensis* for 48 and 96 hs. Data are representative of those found in three independent experiments. No statistically significant differences were detected between BMDM obtained from fresh or frozen BM cells (*P*>0.05).

Next, we investigated if BMDM obtained from fresh or frozen BM cells differ in their ability to support the intracellular replication of pathogens or to clear intracellular microbes. Thus, we used *L. pneumophila*, which is a gram-negative, facultative intracellular bacterium that possesses the ability to replicate within macrophages [18]. Importantly, while wild type *L. pneumophila* is eliminated by C57BL/6 macrophages, mutants deficient for flagellin were shown to bypass macrophage innate immunity and to multiply in macrophages [11], [19], [20]. Thus, we used wild type and *flaA* mutants of *L. pneumophila* to assess the ability of BMDM to restrict or support intracellular microbial replication. We found that BMDM obtained from fresh or frozen BM cells differ neither in their ability to restrict intracellular multiplication of wild type bacteria nor in their ability to support intracellular replication of *flaA* mutant bacteria (Fig. 7A). To further evaluate the intracellular replication of wild type bacteria in macrophages we employed a highly sensitive single cell analysis to evaluate the number bacteria per LCV formed after 9 hours infection in BMDM obtained from fresh or cryopreserved BM cells. We found that the frequency of the number of bacteria per LCV was not altered in BMDM obtained from fresh or frozen BM cells (Fig. 7B). Therefore, these cells did not differ for early restriction of *L. pneumophila* replication in BMDMs. Next, we compared the replication of the pathogenic protozoan parasite *Leishmania (L.) amazonensis* in BMDM obtained from fresh or cryopreserved BM cells. We used a *L. amazonensis* expressing GFP to quantify the parasite replication by FACS. We found that BMDM obtained from fresh and frozen BM cells are equally able to support the intracellular replication of *L. amazonensis* (Fig. 8). To further evaluate cell death induced in response to infection we stained the infected BMDM with propidium iodide and found that infection did not trigger cell permeabilization regardless to the BMDM used (Fig. 8D, E). Collectively, these data indicate that freezing BM cells did not interfere with BMDM functions, such as their ability to eliminate or to support the multiplication of intracellular protozoan and bacterial pathogens.

## Discussion

Over the last two decades, the wide use of transgenic and gene-disrupted mice has had a striking impact on cell biology, microbial pathogenesis and immunology research. Such animals are not only important for in vivo research, but also for in vitro studies performed with primary cells obtained from these mice. Importantly, BMDM obtained from transgenic mice are broadly used as primary cells. Here, we describe a highly efficient method for cryopreservation of murine BM cells for further differentiation into BMDM. We demonstrate that cryopreserved BM can maintain differentiation capacity for at least 6 years. Therefore, this method represents a powerful tool for murine macrophage research, as BMDM can be readily and easily generated from a stock of frozen BM cells.

Preliminary investigation preformed herein indicates that bone marrow-derived dendritic cells can be obtained from cryopreserved BM cells (Fig. 6). Importantly DCs obtained from fresh and cryopreserved BM cells equally respond to LPS as measured by the expression of CD40, CD86 and MHC-II. These preliminary results may indicate that the method described herein may also be useful for dendritic cell research. However, further and extensive characterization of the biology of dendritic cells obtained from fresh or frozen BM cells will be required to allow routine use of dendritic cells from cryopreserved BM cells. As for BMDM, we demonstrate that macrophages obtained from fresh or frozen BM cells do not differ for important macrophage functions such as expression of CD86 and MHC-II in response to LPS or *L. pneumophila* infection and restriction/support of intracellular multiplication of the pathogenic bacteria *L. pneumophila*.

Importantly, the optimizations of the protocol performed herein allow us to use 4×10^6^ BM cells per cryovial, which give rise to about 10 million BMDM per cryovial. Therefore, the sacrifice of a single mouse can generate several BM-containing cryovials (usually from 4 to 10). This valuable material can be maintained frozen for years and further used to perform independent experiments. Finally, the development of the protocol described herein not only saves researchers' time and funds, but also reduces the number of mice sacrificed, which are precious resources often of limited supply.

## References

[1] Gordon S (2007). The macrophage: past, present and future.. Eur J Immunol.

[2] Morrissette N, Gold E, Aderem A (1999). The macrophage–a cell for all seasons.. Trends Cell Biol.

[3] Taylor PR, Martinez-Pomares L, Stacey M, Lin HH, Brown GD (2005). Macrophage receptors and immune recognition.. Annu Rev Immunol.

[4] Balint B, Ivanovic Z, Petakov M, Taseski J, Jovcic G (1999). The cryopreservation protocol optimal for progenitor recovery is not optimal for preservation of marrow repopulating ability.. Bone Marrow Transplant.

[5] Berz D, McCormack EM, Winer ES, Colvin GA, Quesenberry PJ (2007). Cryopreservation of hematopoietic stem cells.. Am J Hematol.

[6] Hiebl B, Fuhrmann R, Franke RP (2008). Characterization of cryopreserved CD14+-human monocytes after differentiation towards macrophages and stimulation with VEGF-A(165).. Clin Hemorheol Microcirc.

[7] Seager Danciger J, Lutz M, Hama S, Cruz D, Castrillo A (2004). Method for large scale isolation, culture and cryopreservation of human monocytes suitable for chemotaxis, cellular adhesion assays, macrophage and dendritic cell differentiation.. J Immunol Methods.

[8] Zamboni DS, Rabinovitch M (2003). Nitric oxide partially controls *Coxiella burnetii* phase II infection in mouse primary macrophages.. Infect Immun.

[9] Englen MD, Valdez YE, Lehnert NM, Lehnert BE (1995). Granulocyte/macrophage colony-stimulating factor is expressed and secreted in cultures of murine L929 cells.. J Immunol Methods.

[10] Berger KH, Isberg RR (1993). Two distinct defects in intracellular growth complemented by a single genetic locus in *Legionella pneumophila*.. Mol Microbiol.

[11] Ren T, Zamboni DS, Roy CR, Dietrich WF, Vance RE (2006). Flagellin-Deficient *Legionella* Mutants Evade Caspase-1- and Naip5-Mediated Macrophage Immunity.. PLoS Pathog.

[12] Feeley JC, Gibson RJ, Gorman GW, Langford NC, Rasheed JK (1979). Charcoal-yeast extract agar: primary isolation medium for *Legionella pneumophila*.. J Clin Microbiol.

[13] Zamboni DS, McGrath S, Rabinovitch M, Roy CR (2003). *Coxiella burnetii* express type IV secretion system proteins that function similarly to components of the *Legionella pneumophila* Dot/Icm system.. Mol Microbiol.

[14] Kram D, Thale C, Kolodziej H, Kiderlen AF (2008). Intracellular parasite kill: flow cytometry and NO detection for rapid discrimination between anti-leishmanial activity and macrophage activation.. J Immunol Methods.

[15] Carregaro V, Valenzuela JG, Cunha TM, Verri WA, Grespan R (2008). Phlebotomine salivas inhibit immune inflammation-induced neutrophil migration via an autocrine DC-derived PGE2/IL-10 sequential pathway.. J Leukoc Biol.

[16] Lovelock JE, Bishop MW (1959). Prevention of freezing damage to living cells by dimethyl sulphoxide.. Nature.

[17] Donnenberg AD, Koch EK, Griffin DL, Stanczak HM, Kiss JE (2002). Viability of cryopreserved BM progenitor cells stored for more than a decade.. Cytotherapy.

[18] Horwitz MA (1983). Formation of a novel phagosome by the Legionnaires' disease bacterium (*Legionella pneumophila*) in human monocytes.. J Exp Med.

[19] Molofsky AB, Byrne BG, Whitfield NN, Madigan CA, Fuse ET (2006). Cytosolic recognition of flagellin by mouse macrophages restricts *Legionella pneumophila* infection.. J Exp Med.

[20] Zamboni DS, Kobayashi KS, Kohlsdorf T, Ogura Y, Long EM (2006). The Birc1e cytosolic pattern-recognition receptor contributes to the detection and control of *Legionella pneumophila* infection.. Nat Immunol.

